# The requirement of IRE1 and XBP1 in resolving physiological stress during *Drosophila* development

**DOI:** 10.1242/jcs.203612

**Published:** 2017-09-15

**Authors:** Huai-Wei Huang, Xiaomei Zeng, Taiyoun Rhim, David Ron, Hyung Don Ryoo

**Affiliations:** 1 Department of Cell Biology, New York University School of Medicine, New York 10016, USA; 2 Key Laboratory of Molecular Biophysics of Ministry of Education, Center for Human Genome Research, College of Life Science and Technology, Huazhong University of Science and Technology, Wuhan 430000, China; 3 Department of Bioengineering, Hanyang University, Seoul 04763, Korea; 4 Department of Clinical Biochemistry, Cambridge Institute for Medical Research, Cambridge CB2 0XY, UK

**Keywords:** Unfolded protein response, Physiological ER stress, *Xbp1*, *Ire1*, Midgut, Gastric caeca, Proventriculus, Accessory gland, Ejaculatory duct, Male sterility

## Abstract

IRE1 mediates the unfolded protein response (UPR) in part by regulating XBP1 mRNA splicing in response to endoplasmic reticulum (ER) stress. In cultured metazoan cells, IRE1 also exhibits XBP1-independent biochemical activities. IRE1 and XBP1 are developmentally essential genes in *Drosophila* and mammals, but the source of the physiological ER stress and the relative contributions of XBP1 activation versus other IRE1 functions to development remain unknown. Here, we employed *Drosophila* to address this question. Explicitly, we find that specific regions of the developing alimentary canal, fat body and the male reproductive organ are the sources of physiological stress that require *Ire1* and *Xbp1* for resolution. In particular, the developmental lethality associated with an *Xbp1* null mutation was rescued by transgenic expression of *Xbp1* in the alimentary canal. The domains of IRE1 that are involved in detecting unfolded proteins, cleaving RNAs and activating XBP1 splicing were all essential for development. The earlier onset of developmental defects in *Ire1* mutant larvae compared to in *Xbp1*-null flies supports a developmental role for XBP1-independent IRE1 RNase activity, while challenging the importance of RNase-independent effector mechanisms of *Drosophila* IRE1 function.

## Abstract (precis)

**Summary:** The stress-response proteins IRE1 and XBP1 are developmentally essential in metazoans. We identify the source of the physiological stress in *Drosophila* and determine the molecular requirements for stress response signaling.

## INTRODUCTION

Endoplasmic reticulum (ER) is a subcellular organelle in eukaryotes where most secretory and membrane proteins are synthesized and undergo folding. Conditions that disrupt or overwhelm the protein-folding capacity of the ER must be resolved to maintain cellular function, and not surprisingly, eukaryotic cells have evolved robust quality control mechanisms that help cells cope with such ER stress. Among those quality control mechanisms is the unfolded protein response (UPR), which refers to signaling pathways that are activated in response to ER stress to regulate gene expression (Walter and Ron, 2011).

A particularly well-characterized branch of the UPR is mediated by IRE1 (also known as ERN1 in mammals) and XBP1, which are conserved across phyla from mammals to *S. cerevisiae* (Walter and Ron, 2011). This pathway is initiated by IRE1, which detects imbalances between unfolded proteins and chaperones through its stress-sensing luminal domain (Aragón et al., 2009; Credle et al., 2005; Gardner and Walter, 2011; Zhou et al., 2006) to activate its cytoplasmic RNase (Korennykh et al., 2011; Lee et al., 2008b). The best-characterized and most important substrate of IRE1 is the XBP1 mRNA, which undergoes an unconventional splicing reaction as part of the UPR. Spliced XBP1 mRNA encodes an active transcription factor isoform, which after translation, promotes the expression of various ER quality control genes (Calfon et al., 2002; Shen et al., 2001; Yoshida et al., 2001).

In addition to this well-established axis of IRE1 signaling, a number of additional regulatory mechanisms associated with IRE1 have been uncovered in cultured cells. These include additional substrates for the RNase activity of IRE1. Unlike XBP1 mRNA, IRE1 cleavage of these other substrates leads to their degradation, a process which is referred to as regulated IRE1-dependent decay (RIDD) (Coelho et al., 2013; Hollien et al., 2009; Hollien and Weissman, 2006). RIDD is believed to lessen the burden of new protein synthesis and folding in the ER, but its functional significance has not been rigorously tested. IRE1 also has an RNase-independent activity in stimulating JNK signaling (Urano et al., 2000). These various cytoplasmic activities of IRE1 are not only activated by misfolded peptides, but also in response to perturbation of lipid balance. Interestingly, IRE1 activation under these conditions occurs even without the luminal domain responsible for detecting misfolded peptides, in yeast as well as in cultured mammalian cells (Promlek et al., 2011; Volmer et al., 2013).

While these mechanisms have been uncovered in cultured cells exposed to exogenously imposed conditions of stress, their roles in dealing with physiological ER stress in metazoan tissues remain poorly understood. It is notable that IRE1α, one of the two mammalian IRE1 genes (also known as ERN1), and XBP1 are developmentally essential in mice (Iwawaki et al., 2009; Lee et al., 2008a; Reimold et al., 2001). However, the reported IRE1-knockout phenotype is different from that of XBP1 knockouts in mice: whereas the developmental lethality of XBP1-knockout embryos has been attributed to liver failure (Lee et al., 2005), the lethality of IRE1α-knockout mice is attributed to a different organ – specifically, the placenta (Iwawaki et al., 2009). Such difference may be due to as yet inexplicable tissue specificity of the two genes, and the functional significance of IRE1-mediated XBP1 activation during normal animal development remains unclear.

Here, we employed the molecular genetic tools of *Drosophila* to understand the precise role of IRE1 and XBP1 signaling during normal metazoan development. The basic mechanism of UPR is conserved in *Drosophila*, and the homologous genes in *Drosophila* are also essential for normal development (Coelho et al., 2013; Ryoo, 2015; Ryoo et al., 2007, 2013). By employing robust stress reporters, we report the identity of tissues that are impacted by the loss of *Ire1* or *Xbp1* during normal development. In addition, we examined the functional significance and the *in vivo* roles of various IRE1 domains through molecular genetic tools. The results indicate that specific tissues require *Ire1* and *Xbp1* during normal development, and the domains of IRE1 involved in the detection of misfolded peptides and splicing of XBP1 mRNA are particularly essential. We also find that developmental phenotypes found upon *Ire1* mutation are more severe than those for *Xbp1*, supporting the idea that IRE1 has XBP1-independent roles. On the other hand, we found no significant evidence that the RNase-independent activities of IRE1 have a role during normal development.

## RESULTS

### Visualization of cells impacted by the loss of *Ire1* or *Xbp1* through a stress-responsive reporter

**Fig. 1.**
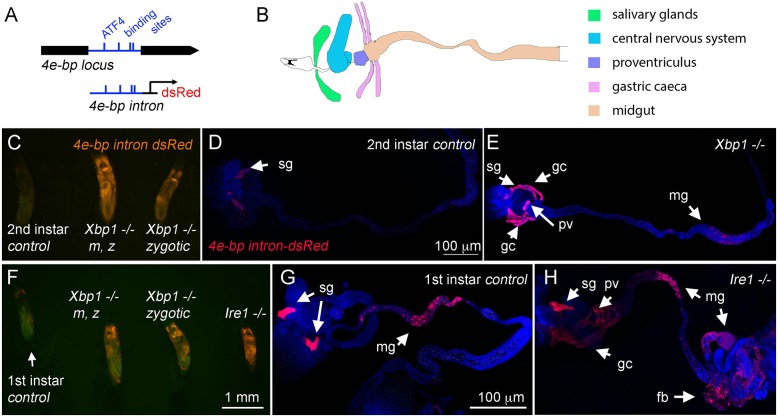
**The ATF4-responsive *4E-BP^intron^ dsRed* reporter is induced in *Xbp1* and *Ire1* mutant larvae.** (A) A schematic diagram of the *4E-BP* locus. The intron is depicted in blue, and the predicted ATF4-binding sites are indicated by vertical lines. The dsRed reporter expression is driven by this intron element (Kang et al., 2017). (B) A schematic diagram of the salivary glands (green), central nervous system (blue), proventriculus (purple), gastric caeca (pink) and midgut (yellow) in the second-instar *Drosophila* larva. Compare this diagram with images in D,E,G and H. (C–H) *4E-BP^intron^ dsRed* expression (red) in the indicated genetic backgrounds. TO-PRO-3 (blue) was used to show the outline of tissues. (C) An intact control of wild-type *Xbp1* (left), an *Xbp1* maternal zygotic (m, z) mutant (center) and an *Xbp1* zygotic mutant (right) at the second-instar stage juxtaposed to each other without dissection. (D) Dissected wild-type second-instar larva with *4E-BP^intron^ dsRed* expression primarily restricted to the salivary glands. (E) In the *Xbp1^ex79−/−^* background, *4E-BP^intron^ dsRed* is strongly induced in tissues beyond the salivary glands, which include the gastric caeca and the proventriculus (arrows). (F) Intact wild-type (left), *Xbp1* maternal zygotic (m, z) mutant (left center), *Xbp1* zygotic mutant (right center) and *Ire1^f02170−/−^* (right) first-instar larvae are juxtaposed. (G) Dissected wild-type first-instar larva showing *4E-BP^intron^ dsRed* expression most prominently in the salivary glands. (H) In the *Ire1^−/−^* larva, the reporter is also strongly expressed in the proventriculus, gastric caeca, midgut and fat body (indicated with arrows). The scale bar in D applies to D and E; the scale bar in F applies to panels C and F, and the scale bar in G applies to images in G and H. sg, salivary glands; gc, gastric caeca; pv, proventriculus; mg, midgut; fb, fat body.

In *Drosophila*, *Xbp1-*null mutants (*Xbp1^ex7−/−^*) fail to develop beyond the second-instar larval stage (Ryoo et al., 2007, 2013). To better understand the basis of this defect, we sought a reporter that could mark cells suffering from the loss of IRE1 and/or XBP1 signaling. We specifically employed a reporter for the PERK (also known as PEK in flies and EIF2AK3 in mammals) and ATF4 (crc in flies) pathway, a branch of the UPR that is parallel to that of IRE1 and XBP1. In cells that require IRE1 and XBP1 signaling for ER homeostasis, it is predicted that the loss of IRE1 or XBP1 would aggravate stress in the ER and thereby hyper-activate PERK and ATF4 signaling. In fact, it had been observed that the loss of one branch of the UPR results in the hyper-activation of the other in mouse tissues that have high secretory burden (Harding et al., 2001). In worms, single mutants of *perk* or *Xbp1* are viable, but the knockdown of both genes result in synthetic lethality, further supporting the compensatory activities of the two pathways (Shen et al., 2001). The PERK and ATF4 reporter that we used is *4E-BP^intron^ dsRed*, which has a cluster of predicted ATF4-binding sites in the 4E-BP intron sequence driving dsRed (Fig. 1A) (Kang et al., 2017).

**Fig. 2.**
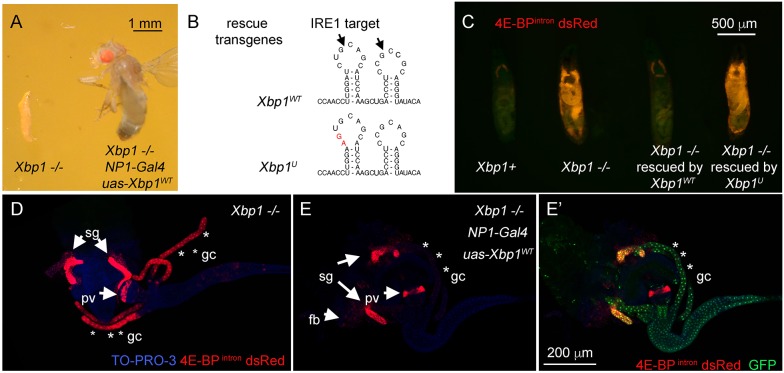
**Rescue of *Xbp1* mutant-associated lethality through the expression of *Xbp1* transgene.** (A) *Xbp1^−/−^* larvae survive only up to the second-instar larval stage (left), whereas *Xbp1* mutants expressing transgenic *Xbp1* with the *NP1-Gal4* driver survive to adulthood (right). (B) The IRE1 target cleavage sequence within the XBP1 mRNA. Arrows point to the sites that are spliced. The wild-type sequence (upper image) and the mutated sequence within the rescue transgene (lower image, in red) are shown. (C–E) The effects of *Xbp1* transgene expression driven by *NP1-Gal4* on the *4E-BP^intron^ dsRed* reporter levels in *Xbp1^−/−^* second-instar larvae (red)*.* (C) Non-dissected second-instar larvae of the indicated genotypes. (D,E) *4E-BP^intron^ dsRed* signal (red) from dissected second-instar larval tissues. TO-PRO-3 (blue) was used to show the outline of tissues. The strong expression of *4E-BP^intron^ dsRed* signal in the *Xbp1^−/−^* background (D) is suppressed by the wild-type *Xbp1* transgene expression through the *NP1-Gal4* driver (E). *UAS-GFP* was co-expressed with the *Xbp1* transgene, and GFP signal (green) marks the *NP-Gal4* active tissues, which includes the gastric caeca (asterisks) and the midgut (E′), and *4E-BP^intron^ dsRed* signal is specifically suppressed in those domains. The scale bar in E′ applies to D and E. sg, salivary glands; gc, gastric caeca; pv, proventriculus; fb, fat body.

A schematic diagram of a few relevant organs in the second-instar larva is shown in Fig. 1B. Specifically, the mouth hook and the salivary glands are at anterior end. They are connected to the esophagus, which passes between the brain lobes to the proventriculus, a bulb-like structure where the ingested food passes through to reach the midgut. Around the proventriculus–midgut junction are four long tubes called the gastric caeca. While the activity of the *4E-BP^intron^ dsRed* reporter is low in the *Xbp1^+^*^/+^ control second-instar larva, the reporter signal was readily visible in the equivalent stage of *Xbp1^ex79−/−^* larvae even without dissection (Fig. 1C, right larva). We also generated a maternal zygotic *Xbp1* mutant larva (Fig. 1C, middle) and found that they also survived to the second-instar larval stage with a similar degree of *4E-BP^intron^ dsRed* reporter activation as in the zygotic mutant. In the *Xbp1^+^*^/+^ background, the reporter activity was mostly confined to the salivary glands, but the dissected *Xbp1* mutants reveal specific *4E-BP^intron^ dsRed* reporter induction in the gastric caeca, proventriculus and certain parts of the posterior intestine (Fig. 1D,E). Fat bodies also induced *4E-BP^intron^ dsRed*, but with lesser intensity (see below in Fig. 2D). Notably, these are the tissues with reportedly high levels of *Xbp1* transcripts (Ryoo et al., 2007, 2013). On the other hand, there were many other tissues that did not show signs of stress in the *Xbp1* mutant background, which included certain parts of the midgut, the larval brain and imaginal discs, indicating that the role of *Xbp1* in suppressing the activation of *4E-BP^intron^ dsRed* reporter in the context of normal fly development is specific to the cell type.

We also examined the effect of *Ire1* loss-of-function mutation on the *4E-BP^intron^ dsRed* reporter. *Ire1* mutants develop only up to the first-instar larval stage, and strong induction of the *4E-BP^intron^ dsRed* in these mutants was also obvious without dissection. The dsRed signal from *Ire1* mutant larvae were visibly more intense than that of zygotic *Xbp1* null mutants as well as maternal zygotic *Xbp1* mutants of the same developmental stage (Fig. 1F). Dissected *Ire1* mutant first-instar larvae showed similar patterns of *4E-BP^intron^ dsRed* induction to that of second-instar *Xbp1* mutants, which included the gastric caeca, several parts of the intestine and the fat body (Fig. 1G,H). These results indicate that the same tissues of the developing larvae are impacted by the loss of *Ire1* or *Xbp1* but the consequences of *Ire1* loss are more severe.

### Rescue of the larval lethality with transgenic *Xbp1*

We found that the larval lethality of the *Drosophila Xbp1* mutants could be rescued by the ectopic expression of *Xbp1* through *tubulin-Gal4* (*tub-Gal4*) and *UAS-Xbp1*. The surviving adults eclosed with the predicted Mendelian ratio (approximately one-third of all progeny, due to the use of balancer chromosomes in the parents that are recessive lethal; Fig. S1). To determine the specific tissue types that require *Xbp1* during development, we expressed *UAS-Xbp1* with tissue-specific Gal4 drivers in *Xbp1* mutants. Various tissue-specific *Gal4* drivers were used (see Materials and Methods), but aside from *tub-Gal4*, only *NP1-Gal4* rescued the larval lethality (Fig. 2A). *NP1-Gal4* is a Gal4 enhancer trap in the Myosin 1A gene (also known as Myo31DF), which encodes a protein that localizes to the brush borders of the gut enterocytes (Morgan et al., 1995). The Gal4 activity is likewise restricted to the enterocytes of the alimentary canal and the salivary gland in the larva (Jiang and Edgar, 2009) (Fig. S2B). In adults, *NP1-Gal4* also marks the intestine, but is not active in tissues such as the ovary (Fig. S2D). Transgenic *Xbp1* expression using this Gal4 driver suppressed signs of ER stress as evidenced by the loss of the *4E-BP^intron^ dsRed* signal (Fig. 2C).

Neither mouse nor fly studies has yet determined the functional significance of IRE1-mediated XBP1 mRNA splicing during development. IRE1 activates XBP1 by cleaving two evolutionarily conserved positions in the double stem-loop structure within the XBP1 mRNA (Calfon et al., 2002; Yoshida et al., 2001). To determine whether IRE1-mediated XBP1 splicing is required for survival, we introduced silent mutations in the *Xbp1* transgene, changing the sequence of a conserved double stem-loop IRE1 target sequence within the mRNA (Fig. 2B). The targeted sequence is conserved from yeast to mammals, and mutation in those sequences impair IRE1-mediated XBP1 mRNA cleavage across phyla (Calfon et al., 2002; Shen et al., 2001; Yoshida et al., 2001). At the same time, the mutations do not alter the encoded amino acid residues, and precede the splice site. Unlike the wild-type transgene, the *UAS-Xbp1* transgene containing such mutated stem-loop (referred to as *UAS-Xbp1^U^*) failed to suppress the *4E-BP^intron^ dsRed* signal in the *Xbp1^−/−^* background (Fig. 2C). Consistently, *Xbp1^U^* expression failed to rescue the larval lethality. Upon dissection of these larvae, we found that the strong *4E-BP^intron^ dsRed* signal in the gastric caeca of *Xbp1* mutants was suppressed by the wild-type *Xbp1* transgene expression through the *NP1-Gal4* driver (Fig. 2D,E). On the other hand, the lower intensity *4E-BP^intron^ dsRed* signal from the fat body was not suppressed, a tissue where *NP1-Gal4* is not active (Fig. 2E). This observation suggests that IRE1 and XBP1 signaling in the alimentary canal is essential for survival during development.

Many other Gal4 drivers that are active in other cell types could not rescue the *Xbp1* lethality when driving *UAS-Xbp1* expression. These included *dilp2-Gal4* driving gene expression in the insulin-producing cells of the larval brain, *prospero-Gal4* that drives gene expression in entero-endocrine cells that specialize in peptide secretion within the midgut, e*scargot-Gal4*, which is expressed in salivary glands, imaginal discs and the adult midgut precursor cells (Fig. S2), the neuronal-specific *Elav-Gal4*, the glia-specific *Repo-Gal4* and the fat body-specific *cg-Gal4.* These observations indicate that *Xbp1* in the nervous system, insulin-producing cells or entero-endocrine cells alone are not sufficient to rescue the developmental lethality associated with the loss of *Xbp1*.

### Requirement for IRE1 during normal development

**Fig. 3.**
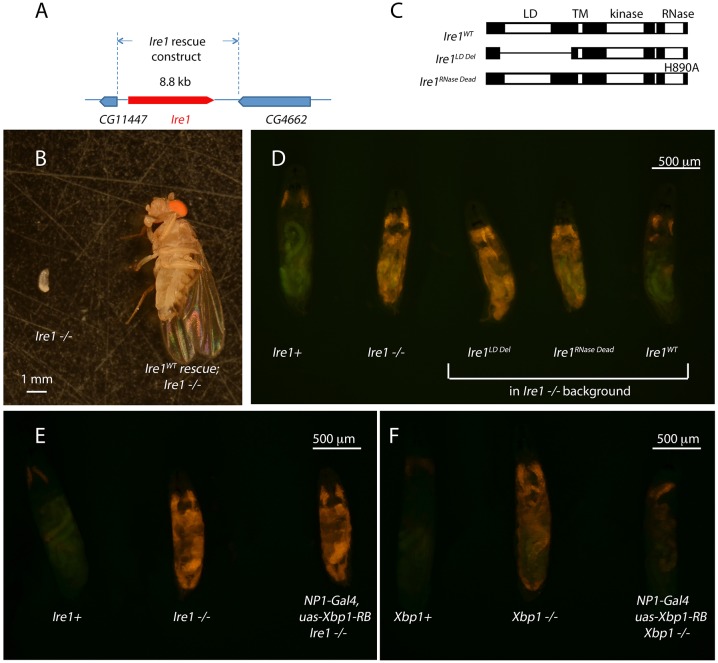
**The roles of specific IRE1 domains and spliced XBP1 in development.** (A) A schematic diagram of the *Ire1* rescue genomic construct. (B) *Ire1^−/−^* larva at its latest stage of survival (left), juxtaposed to an *Ire1^−/−^* fly rescued with the wild-type *Ire1* genomic transgene. (C) A diagram of wild-type and mutant *Ire1* primary structures that were tested for rescue. While the mutations were made in the context of the 8.8 kb genomic rescue transgene, only the changes in the resulting coding sequence are shown here. The mutant constructs are designed to specifically impair misfolded peptide sensing in the ER by the deletion of the luminal domain (*Ire1^LD Del^*) and RNase activity (*Ire1^RNase Dead^*). The latter has a missense mutation that changes H890 to an alanine residue. (D) The effect of the wild-type and mutant *Ire1* transgenes on the *4E-BP^intron^ dsRed* signal of the *Ire1^−/−^* first-instar larvae. The genotypes are indicated below each larva. (E,F) The effect of expressing *Xbp1-RB* (spliced *Xbp1* isoform) through the *NP1-Gal4* driver in the *Ire1* (E), or *Xbp1* (F) mutant backgrounds. All larvae contain the *4E-BP^intron^ dsRed* (red) reporter in the background, and the genotypes are indicated below each larva.

To better understand the developmental role of *Ire1*, we performed a structure function study of IRE1. First, we established that the developmental lethality of the *Ire1^f01270^* allele can be specifically attributed to the loss of *Ire1* by rescuing this lethality with an 8.8 kb genomic transgene from the *Ire1* locus (Fig. 3A,B). The *Drosophila Ire1*-coding sequence is largely conserved with that of yeast and mammalian IRE1, with an ER luminal domain that detects misfolded peptides, and cytoplasmic domains that encode a kinase and an RNase. Through BAC-recombineering of this *Ire1* genomic transgene, we mutated sequences that would abolish specific functions of encoded IRE1 protein (Fig. 3C). Specifically, we expressed an *Ire1^LD Del^* transgene with a deletion in the luminal domain that detects misfolded peptides in the ER lumen. Structure and function analysis of yeast and mammalian IRE1 have shown that deletion of a portion of its luminal domain blocks its ability to respond to misfolded peptides, while not affecting its activation by lipid imbalances (Promlek et al., 2011; Volmer et al., 2013). As deleting a large segment of a protein can compromise overall protein stability, we deleted precisely the part of the *Drosophila* IRE1 that is equivalent to what was deleted in the aforementioned yeast and mammalian studies. To abolish the RNase activity of IRE1, we introduced an H890A mutation, also based on the fact that the equivalent amino acid residue in yeast and mammalian IRE1 is critical for the RNase activity (Korennykh et al., 2009; Lee et al., 2008b). To test whether these mutants abolish IRE1 activity in the larva, we introduced the XBP1–EGFP reporter in which the GFP epitope is designed to be expressed when XBP1 mRNA undergoes IRE1-mediated splicing (Sone et al., 2013). *Ire1^f02170−/−^* with wild-type *Ire1* genomic transgenes readily activated this XBP1–EGFP reporter when exposed to tunicamycin treatment (Fig. S3A,B), but the mutant *Ire1* transgenes failed to do so (Fig. S3C,D). Consistent with these results, these mutant genomic transgenes rescued neither the first-instar larval lethality of *Ire1* nor the strong *4E-BP^intron^ dsRed* signal associated with *Ire1* mutations (Fig. 3D).

If the sole function of IRE1 during development was to splice and activate XBP1 mRNA, the larval lethality of *Ire1* mutants would be rescued by the transgenic expression of the spliced XBP1 isoform, *Xbp1-RB*. We tested this possibility by driving *Xbp1-RB* expression through the *NP1-Gal4* driver, which neither rescued the lethality nor suppressed the strong induction of the *4E-BP^intron^ dsRed* signal in *Ire1* mutants (Fig. 3E). To make sure that the *Xbp1-RB* transgene is functional, we expressed this transgene in the *Xbp1* mutant larva using the *NP1-Gal4* driver, which strongly reduced the *4E-BP^intron^ dsRed* signal (Fig. 3F). These experiments indicate that while IRE1-mediated XBP1 splicing is essential for normal *Drosophila* development, XBP1-independent IRE1 functions are also functionally important.

### Requirements for *Xbp1* beyond the alimentary canal

The rescue of *Xbp1^−/−^* with *NP1-Gal4* and wild-type *UAS-Xbp1* was partially incomplete in a number of ways: the *NP1-Gal4* active domain does not include the proventriculus and the fat body (Fig. 2E′, in green), and consistent with this, stress, as evidenced by a strong *4E-BP^intron^ dsRed* signal, was not resolved in those tissues (Fig. 2E). It was also noticeable that most of the progeny that survived to adulthood were males (Fig. S1). While the males survived close to the Mendelian ratio at 25°C, rescue became very rare at 22°C or lower temperatures. The underlying reasons for such dependence on temperature and sex remain unclear.

**Fig. 4.**
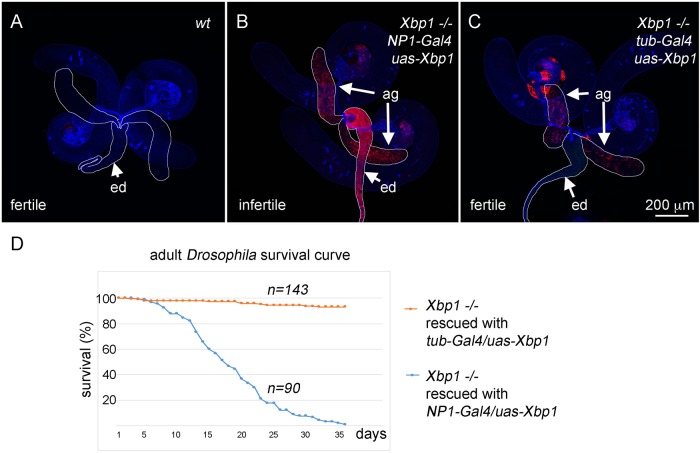
**Requirements for *Xbp1* beyond the alimentary canal.** To examine the effects of *Xbp1* beyond the alimentary canal, *Xbp1* mutants were either rescued to adulthood with an alimentary canal-specific *NP1-Gal4* driver expressing *UAS-Xbp1* (B, and blue line in D), and the phenotypes were compared with mutants that were rescued with a ubiquitous *tub-Gal4* driver (C, orange line in D). (A–C) *4E-BP^intron^ dsRed* (red) in the male reproductive system. Phalloidin labeling is shown in blue. Ejaculatory ducts (ed) and accessory glands (ag) are outlined and indicated with arrows. (A) In the wild-type background, the reporter expression level is insignificant (genotype, *4E-BP^intron^ dsRed/+*). (B) *NP1-Gal4*-rescued mutants have male infertility, and correlating with this, strong reporter signals are detected in the ejaculatory ducts and accessory glands. (C) *Xbp1* mutants rescued with *tub-Gal4* are fertile, and correlating with this, there is reduced reporter activity in the ejaculatory duct. (D) Lifespan of *Xbp1* mutant adult flies that were rescued with either *tub-Gal4* (red) or *NP1-Gal4* (blue) driving *UAS-Xbp1*. The number of flies examined are indicated as *n* on the graph. The scale bar in C also applies to panels A and B.

In adults, we found that the rescued males were completely sterile. To determine the specific tissues affected in these adults, we examined *4E-BP^intron^ dsRed* expression in the male reproductive system. The reporter was largely inactive in the wild-type background, whereas strong signs of stress were detected through the reporter in the accessory glands and ejaculatory ducts of *Xbp1* mutants that were rescued to adulthood with *NP1-Gal4* and *UAS-Xbp1* (Fig. 4A,B). When the ubiquitous *tub-Gal4* driver was used instead of *NP1-Gal4*, such conditions rescued not only larval lethality, but also male infertility. Correlating with the restoration of fertility, *tub-Gal4-*mediated rescue suppressed *4E-BP^intron^ dsRed* induction in the ejaculatory duct (Fig. 4C). In addition to these defects, *Xbp1* mutants that had been rescued with *NP1-Gal4/UAS-Xbp1* had significantly shorter lifespans (Fig. 4D). These observations suggest that *Xbp1* plays functionally significant roles in tissues beyond the alimentary canal.

### *Xbp1* mutant larvae show hyperactive innate immune response

**Fig. 5.**
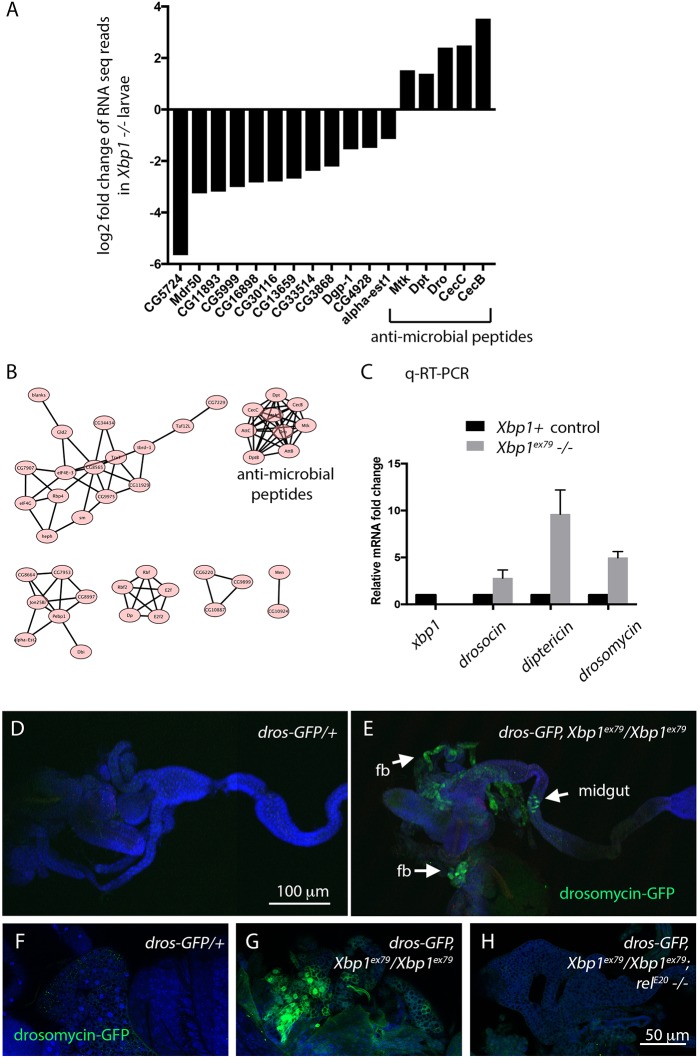
**The innate immune response is activated in *Xbp1* mutant larvae.** (A) A select set of genes with altered gene expression (adjusted *P* value <0.05) in *Xbp1* mutant larvae. The full list is in Table S1. The *y*-axis shows the log2 fold change of RNAseq reads in *Xbp1* mutants. Genes shown here are those previously reported to be tunicamycin-inducible genes in the fly (reported in Chow et al., 2013; left 12 bars), and those encoding anti-microbial peptides (right five bars). (B) Network analysis of the genes that are induced in *Xbp1* mutants. Those that encode anti-microbial peptides form a major cluster. (C) Validation of AMP transcript induction through qRT-PCR. qRT-PCR against *Xbp1* was performed to confirm that the samples were from *Xbp1^ex79−/−^* null larvae. As representative AMPs, *drosocin*, *diptericin* and *drosomycin* transcripts were examined*.* (D,E) *drosomycin-GFP*, a reporter for an anti-microbial peptide expression (green) in wild-type (D) and *Xbp1* mutant (E) second-instar larvae. TO-PRO-3 (blue) was used to show the outline of tissues. D and E are composite images.  The scale bar for D and E is shown in D. (F–H) Induction of *drosomycin-GFP* in the fat body of *Xbp1* mutants (wild-type in F and *Xbp1^−/−^* in G) is abolished in the mutant background of *rel^20^* (H), which encodes a Rel family of immune responsive transcription factor*.* The scale bar for F–H is shown in H.

To obtain an unbiased view of the changes in gene expression that occurs in *Xbp1* mutant larvae, we performed RNAseq analysis of transcripts from *Xbp1^+/+^* control and *Xbp1^ex79−/−^* second-instar larvae. The full list of analyzed RNAs, and their relative levels in mutant versus wild-type are shown in Table S1. The entire sequence data is accessible through NCBI's Gene Expression Omnibus (GEO accession number GSE99676). The transcripts from the RNAseq data were ranked by the adjusted *P* values (<0.05) in order to select significantly upregulated or downregulated genes in *Xbp1* mutants for further analysis. According to this criteria, 79 genes were downregulated in *Xbp1* mutants, while 75 genes were upregulated (see Table S1). The gene ontology (GO) terms with *P<*0.05 are listed in Tables 1 and 2. Specifically, the downregulated gene GO terms included those involved in dephosphorylation (GO 0016791, *P* value of 3.5×10^−3^), oxidation-reduction (GO 0055114, *P* value of 4.2×10^−3^) and iron ion binding (GO 0005506, *P* value of 4.3×10^−2^). Some of the genes were previously reported as tunicamycin-inducible genes in flies (Chow et al., 2013; Fig. 5A), consistent with the idea that their expression depends on *Xbp1.* Prominent among the genes whose expression increased significantly were those encoding anti-microbial peptides (AMPs) (GO 0019731, *P* value of 2.40×10^−5^), which included *cecB*, *cecC*, *mtk*, *dro* and *dipt* (also known as *DptA*) (Fig. 5A, Table 2). Induction of AMP transcripts in *Xbp1^−/−^* larvae were further validated through quantitative real-time PCR (qRT-PCR; Fig. 5C). To visualize the pattern of AMP induction, we utilized *drosomycin-GFP*, a reporter that is driven by the upstream regulatory element of one of the AMPs (Ferrandon et al., 1998). *Xbp1* mutant larvae had strong *drosomycin-GFP* induction, most prominently in the fat body, but also occasionally in the midgut epithelium (Fig. 5D,E). There is no evidence that these innate immune response genes are under direct transcriptional control by XBP1. Rather, innate immune response activation may be a secondary effect of tissue damage and impairment due to the loss of *Xbp1*. In fact, *drosomycin-GFP* induction was strongly suppressed when the canonical innate immune response was blocked in a *rel* mutant background (Fig. 5F–H), which disrupts a *Drosophila* NF-κB homolog that is a key mediator of the innate immune pathway (Hedengren et al., 1999). The association between the loss of *Xbp1* and innate immune response had been reported in other organisms, such as mice (Adolph et al., 2013; Kaser et al., 2008). In *C. elegans*, *Xbp1* mutants become susceptible to *Pseudomonas aeruginosa* infection (Richardson et al., 2010). Thus, these observations reveal a correlative relationship between impaired UPR and active innate immune response.

**Table 1.**
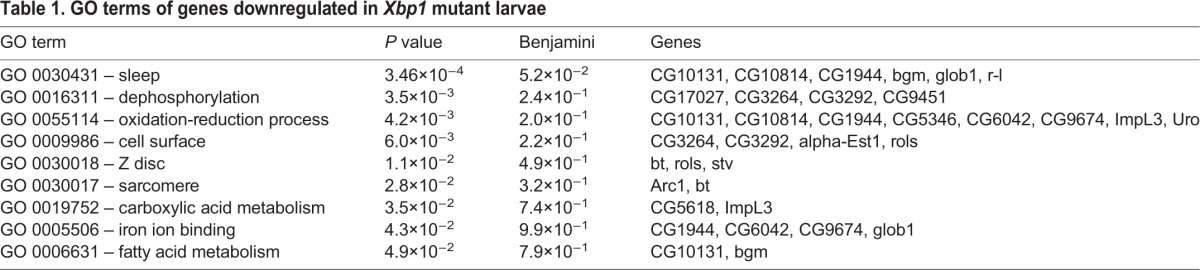
**GO terms of genes downregulated in *Xbp1* mutant larvae**

**Table 2.**
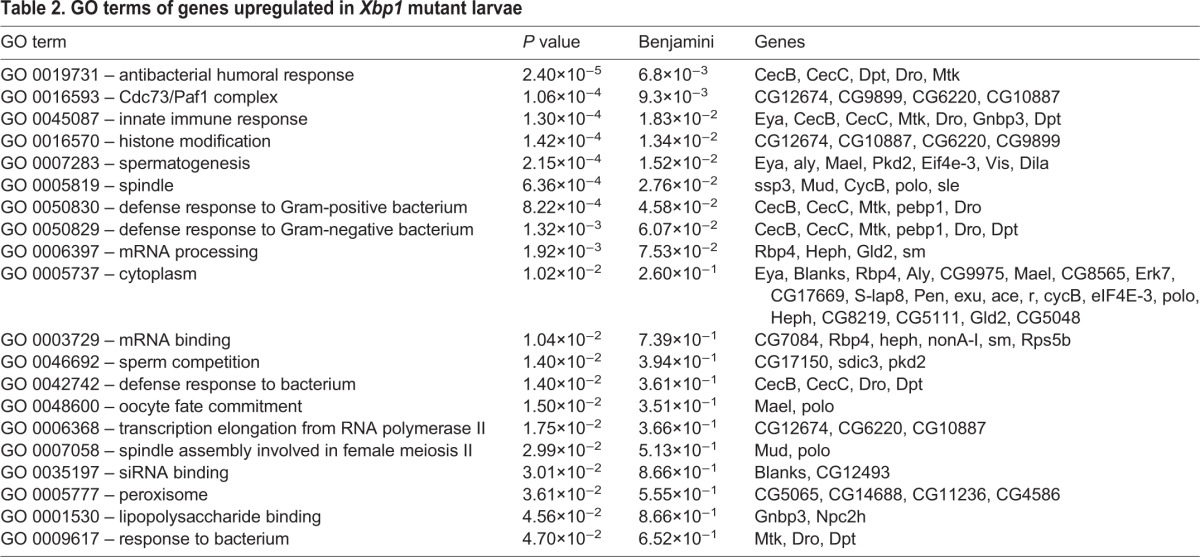
**GO terms of genes upregulated in *Xbp1* mutant larvae**

## DISCUSSION

Here, we report evidence that IRE1 plays essential developmental roles through its XBP1-dependent and -independent activities during normal *Drosophila* development and tissue homeostasis. While it was known that IRE1α and XBP1 are developmentally essential in mice (Iwawaki et al., 2009; Kaser et al., 2008; Lee et al., 2008a; Reimold et al., 2001), our study is the first to determine the functional significance of IRE1-mediated XBP1 splicing versus XBP1 independent function during metazoan development. The results show that this branch of UPR is engaged in resolving physiological stress associated with normal development, and the ability of IRE1 to splice XBP1 mRNA plays a particularly prominent role during this process.

In addition to the IRE1 and XBP1 axis, several cell culture studies have revealed XBP1-independent roles of IRE1. One of these is a role for IRE1 in degrading mRNAs other than XBP1 (Hollien et al., 2009; Hollien and Weissman, 2006). Recently, the significance of such a mechanism has been validated *in vivo*, specifically in the developing *Drosophila* eye (Coelho et al., 2013). In addition, IRE1 regulates JNK signaling that is independent of the RNase activity of IRE1 (Urano et al., 2000); however, the significance of that signaling axis during metazoan development has not yet been determined. Consistent with the XBP1-independent role of IRE1, we also find that the *Ire1* loss-of-function phenotype is generally stronger in the larva than that of *Xbp1* mutants, specifically in terms of earlier lethality and stronger induction of the *4E-BP^intron^ dsRed* reporter. Our structure–function study provides further hints regarding the two XBP1-independent roles of IRE1: an RNase-dependent process of degrading other mRNAs (RIDD) and an RNase-independent process of JNK signaling. Our data particularly highlights the importance of RNase-dependent IRE1 function, as the RNase-dead allele of *Ire1* fails to rescue the first-instar larval lethality and as the degree of *4E-BP^intron^ dsRed* induction is indistinguishable from that of *Ire1* nulls.

In this study, the use of the *4E-BP^intron^ dsRed* reporter has allowed the visualization of specific developing tissues that are impacted by the loss of *Ire1* and *Xbp1.* The results indicate that the midgut, and in particular, the gastric caeca are particularly affected by the loss of IRE1 and XBP1 signaling. Such observations were somewhat unexpected, as there has been no literature implicating gastric caeca and proventriculus with higher physiological stress in the ER, and in adult *Drosophila*, knockdown of *Ire1* shows a rather mild phenotype in triglyceride metabolism and a modest change in adult lifespan (Luis et al., 2016). In fact, not much is known about the precise role of the developing gastric caeca. A small number of digestive enzymes that have been characterized in *Drosophila* are expressed in the gastric caeca and proventriculus (Grönke et al., 2005; Matsumoto et al., 1995). Taken together, these observations raise the possibility that the gastric caeca is a major exocrine organ in *Drosophila*, perhaps playing a role similar to that of the mammalian pancreas in synthesizing and secreting digestive enzymes. We speculate that the essential requirements of *Ire1* and *Xbp1* in the alimentary canal are due to their roles in dealing with physiological levels of ER stress caused by its high secretory protein levels.

Outside the alimentary canal, we find that the accessory glands and the ejaculatory duct of male reproductive organs are particularly sensitive to the loss of *Xbp1*. This is consistent with our understanding that the accessory gland is a secretory organ that produces seminal fluid proteins (SFPs) (Avila et al., 2011; Monsma et al., 1990). Consistent with this, we had reported previously that this organ shows inherent IRE1 activity as evidenced by the XBP1–GFP-based splicing reporter (Sone et al., 2013). A different reporter for *Xbp1* expression in *Drosophila* specifically marks the accessory gland and the ejaculatory duct (Ryoo et al., 2013). Furthermore, a recent study has found that the accessory gland is particularly vulnerable to conditions that perturb ER protein folding (Chow et al., 2015). The results reported in this study show that these tissues are not only vulnerable to exogenously imposed stress, but also have physiological ER stress that requires *Xbp1* for resolution.

In summary, results presented in this study establish the importance of IRE1 and XBP1 signaling in a number of normally developing tissues with no experimentally imposed stress. The results suggest that cells suffer from physiological ER stress as part of the normal developmental program that needs to be resolved through the IRE1 and XBP1 pathway. On the other hand, we do not find evidence that the unconventional roles of IRE1 that are independent of its RNase and luminal domains are necessary during development.

## MATERIALS AND METHODS

### Constructs

*UAS-Xbp1-RA* was generated by cloning the entire *Xbp1-RA* coding sequence into the pUAST plasmid. These constructs were injected into flies using the standard P-element transgenesis technology. *UAS-Xbp1^U^* was generated through site-directed mutagenesis of the conserved splice site residues that introduced silent mutations, as shown in Fig. 4A.

*Ire1* genomic rescue constructs were generated by cloning the 8.8 kb *Ire1* genomic locus through recombineering. Specifically, the sequence between two adjacent genes, CG11447 and CG4662, was cloned from a BAC clone into the P[acman] plasmid using established recombineering protocols (Venken et al., 2006). Subsequently, five tandem repeats of the HA epitope tag were introduced after IRE1 residue 506 using GalK-based recombineering. The epitope tag did not interfere with IRE1 function, as this transgene was able to rescue the lethality of the *Ire1^−/−^* mutants. IRE1^LD Del^ was made by deleting the sequence that encodes the amino acid residues from amino acids 55 to 381. IRE1^RNase Dead^ had the H890 replaced by an alanine residue. These P[acman] constructs were injected into flies to generate transgenic lines by using the phi31-integrase, targeted to the chromosomal location 51F.

### Fly genetics

*Xbp1^ex79^* (Coelho et al., 2013), *Ire1^f02170^* (Kang et al., 2012), *tubulin-Gal4* (Lee and Luo, 1999), *dilp2-Gal4* (Rulifson et al., 2002), *dcg-Gal4* (Arsham and Neufeld, 2009), *NP1-Gal4* (Jiang and Edgar, 2009), *cadudal-Gal4* (Ryu et al., 2008), *drosomycin-GFP* (Ferrandon et al., 1998) and *4E-BP^intron^ dsRed* (Kang et al., 2017) were described previously. The *Xbp1^ex79^* allele has a deletion from 260 bp upstream to 748 bp downstream of the start codon, which includes regions that encode the DNA-binding domain and the IRE1-mediated mRNA splice site (Coelho et al., 2013), and the *Ire1^f02170^* allele has a transposon insertion within the coding sequence. The *Ire1^f02170^* line obtained from the Bloomington Stock Center had background lethal mutations, which were cleaned up by backcrossing to the Ryoo laboratory *w^1118^* stock. Such backcrosses resulted in the isogenization of the *Ire1^f02170^* with other alleles generated in the Ryoo laboratory, including *Xbp1^ex79^*. The effects with *UAS-Xbp1* transgenes were validated with at least two independent lines, which gave consistent results. To generate maternal *Xbp1* mutants, we rescued *Xbp1^−/−^* flies by re-introducing transgenic *Xbp1* through *NP1-Gal4/UAS-Xbp1.* As shown in Fig. S2, *NP1-Gal4* is not active in the female ovary, and therefore, generates *Xbp1^−/−^* mothers that cannot deposit *Xbp1* mRNA into the oocyte. These females were crossed to *4E-BP^intron^ dsRed*, *Xbp1/CyO* males. Half of the progeny were maternal zygotic *Xbp1* mutants (the other half were CyO-containing *Xbp1^+/+^* flies), which were distinguishable through the strong *4E-BP^intron^ dsRed* signal visible at the second-instar larval stage.

### Survival assays

To assess the degree of *Xbp1^−/−^* rescue by Gal4/UAS-mediated transgene expression, crosses were set up with ten males and ten females and the cross was transferred to fresh food every 2 days. The progenies were allowed to develop at 25°C until they reached adulthood. Based on the scheme of the cross, one-third of the flies were expected to have the *Xbp1^−/−^* genotype if all flies were to survive equally. For adult survival assays, ∼20 adult flies were reared in each vial and reared at 25°C. The flies were passed to fresh food every 2 days and the number of dead flies were counted at that point.

### Immunohistochemistry

The following antibodies were used: rabbit anti-GFP (Invitrogen cat. no. A-6455, 1:500). In addition, TO-PRO-3 (Fisher cat. #T3605) and phalloidin conjugated to Alexa Fluor 647 (Invitrogen cat. no. A22287) were used to visualize nuclei of cells and actin filaments. The antibody labeling was done under the standard conditions, which included 20 min of fixation in 4% paraformaldehyde solution, and subsequent incubation with antibodies in PBS that contained 0.2% Triton X-100.

### RNAseq and bioinformatics analysis

RNA was isolated from second-instar larvae (48 h old) of *Xbp1^ex79−/−^* and *Xbp1^+/+^* genotypes. As an *Xbp1^+/+^* control, a precise excision line from *Xbp1^CB02061^* was used. The latter is the line that was also used to derive an imprecise excision allele *Xbp1^ex79^* (Coelho et al., 2013). Two independent extractions of RNAs from each genotype were purified through Qiagen RNeasy columns. RNAseq libraries were prepared using the Illumina TruSeq Stranded Total RNA library prep, with Ribozero Gold, starting from 500 ng of DNase I-treated total RNA, following the manufacturer's protocol, with the exception that 13 cycles of PCR were performed to amplify the libraries, to keep the duplication rate lower than with the recommended 15 cycles. The amplified libraries were purified using AMPure beads, quantified by Qubit and qPCR, and visualized in an Agilent Bioanalyzer. The libraries were pooled equimolarly, and loaded at 8 pM, on a high output HiSeq 2500 flow cell, as paired 50 nucleotide reads for sequencing at the NYU Langone Genome Technology Center. The sequencing reads were aligned to the dm6 reference genome using the Tophat/2.1.1 sequencing analysis package, and HTC count 0.6.1 was used to generate raw gene counts. The package DESeq2 was used for differential gene analysis. The full results have been deposited in NCBI's Gene Expression Omnibus (GEO) and are accessible through GEO series accession number GSE99676. The Database for Annotation, Visualization and Integrated Discovery (DAVID) was used for functional annotation (http://david.abcc.ncifcrf.gov/) of genes whose expression changed significantly in *Xbp1* mutants (*P*<0.05). Network analysis for the selected genes was performed using The Search Tool of the Retrieval of Interacting Genes (STRING) databases (http://string-db.org). Analyzed network and expression level of individual genes were visualized by Cytoscape software (ver 3.4.0).

### Quantitative real-time PCR

Total RNA was extracted from 48-h-old larvae using TRIzol reagent (Invitrogen). From this, single-stranded cDNA was synthesized by using the Superscript III reverse transcript kit (Thermo Fisher Scientific). Real-time PCR was performed by using a Power SYBR Green PCR master mix (Thermo Fisher Scientific). The following primers were used for RT-PCR: Xbp1 forward, 5′-CGTCGAACATGGATGACGATAA; Xbp1 reverse, 5′-GAGTCCAGCTTGTGGTTCTT-3′; CecA1 forward, 5′-GGGTGGCTGAAGAAAATTGG-3′; CecA1 reverse, 5′-ACATTGGCGGCTTGTTGAG-3′; CecA2 forward, 5′-TGGCAAGAAAATCGAACGTG-3′; CecA2 reverse, 5′-CTCGAGCAGTGGCTGCAA-3′; CecB forward, 5′-CGTCTTTGTGGCACTCATCC-3′; CecB reverse, 5′-CCTGGTATGCTGACCAATGC-3′; CecC forward, 5′-CCGGTTGGCTGAAGAAACTT-3′; CecC reverse, 5′-TCCCAGTCCTTGAATGGTTG-3′; Drososin forward, 5′-GTTTTCCTGCTGCTTGCTTG-3′; Drososin reverse, 5′-GGCAGCTTGAGTCAGGTGAT-3′; Drosomycin forward, 5′-TGCCTGTCCGGAAGATACAA-3′; Drosomycin reverse, 5′-CTCCTCCTTGCACACACGAC-3′; *crc* forward, 5′-AAAACCCGTGCTCGTAAAGG-3′; and *crc* reverse, 5′-CGAGCTCCTTAGCACGCATA-3′. The RT-PCR counts with these primers were normalized to that of *Drosophila* Ribosomal protein L15. Primer sequences to amplify the latter were as follows: Rpl15 forward, 5′-AGGATGCACTTATGGCAAGC-3′ and Rpl15 reverse, 5′-GCGCAATCCAATACGAGTTC-3′.

### Statistics and bioinformatics analysis

To calculate the statistical significance of qRT-PCR results, experiments were repeated three times and the results were subjected to *t*-test analysis. The *n* number for the total number of flies subjected to lifespan examination in Fig. 4D are indicated in the graph (*n=143* for *tubulin-Gal4* rescued flies, and *n=90* for *NP1-Gal4* rescued flies).
